# Characterization of Fluorescein Arsenical Hairpin (FlAsH*)* as a Probe for Single-Molecule Fluorescence Spectroscopy

**DOI:** 10.1038/s41598-017-13427-8

**Published:** 2017-10-12

**Authors:** Dennis D. Fernandes, Jasbir Bamrah, Senthilkumar Kailasam, Gregory-Neal W. Gomes, Yuchong Li, Hans-Joachim Wieden, Claudiu C. Gradinaru

**Affiliations:** 1 Department of Physics, University of Toronto, Toronto, Ontario, M5S 1A7 Canada; 2 Department of Chemical & Physical Sciences, University of Toronto Mississauga, Mississauga, Ontario, L5L 1C6 Canada; 3 Alberta RNA Research & Training Institute, Department of Chemistry & Biochemistry, University of Lethbridge, Lethbridge, Alberta T1K 3M4 Canada

## Abstract

In recent years, new labelling strategies have been developed that involve the genetic insertion of small amino-acid sequences for specific attachment of small organic fluorophores. Here, we focus on the tetracysteine FCM motif (FLNCCPGCCMEP), which binds to fluorescein arsenical hairpin (FlAsH), and the ybbR motif (TVLDSLEFIASKLA) which binds fluorophores conjugated to Coenzyme A (CoA) via a phosphoryl transfer reaction. We designed a peptide containing both motifs for orthogonal labelling with FlAsH and Alexa647 (AF647). Molecular dynamics simulations showed that both motifs remain solvent-accessible for labelling reactions. Fluorescence spectra, correlation spectroscopy and anisotropy decay were used to characterize labelling and to obtain photophysical parameters of free and peptide-bound FlAsH. The data demonstrates that FlAsH is a viable probe for single-molecule studies. Single-molecule imaging confirmed dual labeling of the peptide with FlAsH and AF647. Multiparameter single-molecule Förster Resonance Energy Transfer (smFRET) measurements were performed on freely diffusing peptides in solution. The smFRET histogram showed different peaks corresponding to different backbone and dye orientations, in agreement with the molecular dynamics simulations. The tandem of fluorophores and the labelling strategy described here are a promising alternative to bulky fusion fluorescent proteins for smFRET and single-molecule tracking studies of membrane proteins.

## Introduction

In recent years, there has been a rapid increase in both the development and utilization of fluorescence probes as molecular reporters for protein dynamics in cellular biology^1^. Fusion-tag proteins, such as green fluorescent proteins (GFP), acyl carrier proteins (ACP), SNAP-, Halo- and CLIP-tags, have grown in popularity because they can be inserted at the genetic level of the protein of interest (POI) at either the N- or C-terminus^2–7^. Fluorescent proteins (FPs) have their chromophores buried within a beta-barrel folded structure, whereas the fusion methods place synthetic fluorophores onto active sites near the surface of the fusion-tag when attached to the POI, and are often facilitated by biochemical catalytic conditions^2,3,8,9^. A variety of FRET sensors were developed using FP colorimetric variants and fusion-tags to probe complex cellular processes^1,10,11^. These studies include post-translational modifications, conformational changes, metal-ion binding and protein-protein interactions^2^. FP variants however, are less bright and photostable than synthetic fluorophores (e.g., AlexaFluor® dyes) and are known to show large variations in brightness due to differential chromophore maturation^2,12^.

Fusion-tags can alleviate this concern through the incorporation of high-quality fluorophores, however, drawbacks pertaining to bulkiness and steric hindrance remain. These factors limit both the kinetic and spatial resolution^9^. Fusion-tag proteins are typically comprised of 77–300 amino acids (~8 to 30 kDa), which, depending on the insertion point, could result in loss of functionality of the tagged protein^7,8^. Consequently, genetic-insertion of these probes have been limited to either the N- or the C-terminus, restricting the dynamical and structural information that can be accessed. Short peptide motifs consisting of less than 12 amino acids have recently been developed to bind covalently to synthetic organic fluorophores, which can be inserted genetically into flexible loops within proteins^13,14^. These motifs are potentially less disruptive to the biological function than bulky FPs or fusion-tag proteins. Peptide-based labelling methods can be categorized into three broad subgroups: (a) small molecule-dependent recognition, (b) enzyme-mediated labelling and (c) labelling via peptide–peptide interactions^15^. The two peptide motifs considered for this study are the enhanced tetra-cysteine FCM motif (FLNCCPGCCMEP)^16^ and the ybbR motif (TVLDSLEFIASKLA)^14^.

The FCM motif has become a highly advantageous tool in molecular biology, as it provides site-specific rigid covalent binding of bisarsenical-fluorophore analogs onto the POI^17^. The two most commonly used bisarsenical-fluorophores are fluorescein arsenical hairpin (FlAsH), and resorufin arsenical hairpin (ReAsH)^1^. A series of tetracysteine motif variants, have been used to study a wide range of biological systems, ranging from pathogenic effectors in bacterial cells to β-tubulin in yeast cells^18^, and even mammalian G protein-coupled receptors (GPCRs)^19–21^. Predominantly, this method of labelling has been used in combination with FP-variants of the POI, in order to obtain dynamic information regarding changes in protein conformation or protein-protein association by monitoring changes in FRET in live cells^19,22^. Many of these studies however, placed little attention on characterizing and understanding signal variations due to photophysical processes of the fluorophores, which can lead to misinterpretation of data.

The photophysical properties of bisarsenical fluorophores have not been studied in detail and therefore their suitability as single-molecule (sm) fluorescent probes has yet to be assessed. This information will not only be useful for designing smFRET experiments using these versatile fluorescent tags, but also for disentangling protein fluctuations from photophysics dynamics (*i.e*., photoblinking) in fluorescence quenching experiments (e.g., FCS, lifetime)^23^. Fluorophores that are excited with blue-green light are typically susceptible to quenching by native amino acids (*i.e*., tyrosine, tryptophan, histidine, methionine, etc.). Depending on their proximity to the fluorophore, these residues can induce variations in the emission intensity and also decrease the fluorescence lifetime^24^. The process is known as Photoinduced Electron Transfer (PET) and the resulting intensity fluctuations are a good proxy for fast protein conformational dynamics on the 20–200 ns timescale^25,26^. Fluorescence Correlation Spectroscopy (FCS) is sensitive to intensity fluctuations on the nanosecond-millisecond range and is therefore an appropriate method to measure the rate of these photodynamic conformational transitions^27,28^. This type of FCS analysis is known as PET-FCS^29^. To be a promising candidate for PET-FCS, a fluorophore must exhibit “silent” intrinsic photophysics on the relevant timescale (*i.e*., nanosecond-to-microsecond)^28^. AlexaFluoro®488 has been shown to be a good candidate for monitoring PET in proteins, due to its well-behaved and controllable photophysical nature within the PET-FCS dynamic range of analysis^30^. For bisarsenical fluorophores the relevant photophysics has not been studied well, and the potential use of these probes for PET studies still has to be assessed.

Time-resolved fluorescence anisotropy is commonly used to measure the global motion of proteins, as well as local, segmental fluctuations of the polypeptide chain on the nanosecond timescale^31–33^. To date, the majority of fluorescence studies of proteins have utilized single-cysteine mutants conjugated to maleimide-linked-fluorophores^34^. The linker between the maleimide group and the fluorophore typically consists of 5–6 saturated carbon-carbon bonds, which amounts to a length ranging from ~0.5 Å to 10 Å. This configuration leads to the convolution of the faster segmental dynamics of the POI with the dynamics of the geometrically flexible linker. Thus, the presence of long linkers between the POI and the fluorophore tag complicates the estimation of the kinetic rates of local conformational fluctuations as estimated from fitting the anisotropy decay data. In contrast, FlAsH attaches rigidly, without a linker group, directly onto the protein backbone. This configuration is more favorable than the classic labelling approaches used to simultaneously resolve the local dynamics and the global rotation of the POI.

The ybbR motif binds fluorophores conjugated to Coenzyme A (CoA) onto the active serine site near the N-terminus in the presence of the enzyme 4′-phosphopantetheinyl transferase (Sfp synthase), as described previously^11,35^. This approach also has been used to study an array of biological systems, from FRET measurements to tracking of membrane receptors, including transferrin receptors (TfRs) and GPCRs, in live cells^9,11,14^. In addition to providing a means for labelling POIs with fluorophores of choice, this labeling method is also advantageous in that the CoA-conjugated fluorophores have a longer linker (~10 Å)^9^, which can prove useful when they act as acceptors for a rigidly bound donor fluorophore. Typically, FRET experiments with FlAsH-labelled POIs have been paired with either its red-shifted counterpart, ReAsH, or fairly rigid FP-mutants. A freely-rotating acceptor fluorophore, however, can help preserve the assumption that the angles between the donor and acceptor emission dipoles remain randomly oriented, and is numerically expressed as the orientation factor (κ^2^)^36^. This assumption of isotropic dipole orientation (*i.e*., κ^2^ = 2/3) might have not been satisfied in previous FRET studies using FlAsH-FCM and FP tags, which are slow to reorient on the timescale of fluorescence emission^21^. Thus, for an accurate interpretation of smFRET data, an estimation of κ^2^ is key and can be estimated experimentally or using molecular dynamic simulations which was used in this study^37^.

Here, we designed a peptide with both motifs, FCM and ybbR, as a proof-of-concept for orthogonal labelling for smFRET experiments. Typically, FRET requires the design of double-cysteine mutants of the POI and relies on stochastic labelling with maleimide-conjugated fluorophores^38^. This competitive labelling approach results in a mixture of singly-labelled and doubly-labelled POIs, whereby the location of the donor or the acceptor fluorophore could be interchanged. If mutation sites are not chosen carefully, the local environment of the two probes could affect the measured FRET efficiency and complicate the data interpretation^34^. Site-specific labelling of the donor and the acceptor fluorophores uniquely determines the local environment of the two probes and eliminates the above-mentioned complexities of the smFRET data analysis. Orthogonal labelling approaches may not completely eliminate donor-only or acceptor-only labelled molecules in the final sample, however, these species can be resolved and isolated during analysis using alternating laser excitation (ALEX)^39^.

Our test peptide (FCMybbR) contains a hexahistidine tag (His_6x_) at the N-terminus followed by the FCM and the ybbR motifs (HHHHHHGFLNCCPGCCGTVLDSLEFIASKLA). This design allowed us to characterize FlAsH as a probe for PET-FCS studies for resolving fast segmental conformational dynamics, and as a donor for smFRET measurements with the CoA-AF647 acceptor. Molecular dynamic (MD) simulations explored the conformational space of the FCMybbR peptide over a 300-ns time span with/without fluorophore tags. The computational data delivered information on the timescale of structural fluctuations, the solvent-accessibility of each labelling motif, the structural-stability upon attachment of the fluorophores and the distribution of distances between FlAsH and AF647 when bound to the peptide, along with information pertaining to the dye-pairs’ orientation factor. The fluorescence lifetimes, the anisotropy decay and the correlation decay lifetimes of FlAsH-FCMybbR were measured in bulk and the results were interpreted in terms of the fast (sub-μs) conformational fluctuations and the global dimensions of the peptide. Furthermore, efficient *in-situ* orthogonal-labelling of FlAsH-FCMybbR with AF647 was confirmed at the single-molecule level via TIRF imaging. Multiparameter smFRET-burst data provided more detailed information on the photophysics of FlAsH (*i.e*., the distributions of fluorescence lifetimes and molecular brightness), as well as information on the size of the peptide which was corroborated with MD simulations. This study provides a detailed description of the photophysical properties of FlAsH and demonstrates the suitability of this class of bisarsenical fluorophores for single-molecule fluorescence studies of structural dynamics of proteins.

## Results and Discussion

### Peptide design for orthogonal FlAsH and CoA labelling

**Figure 1:**
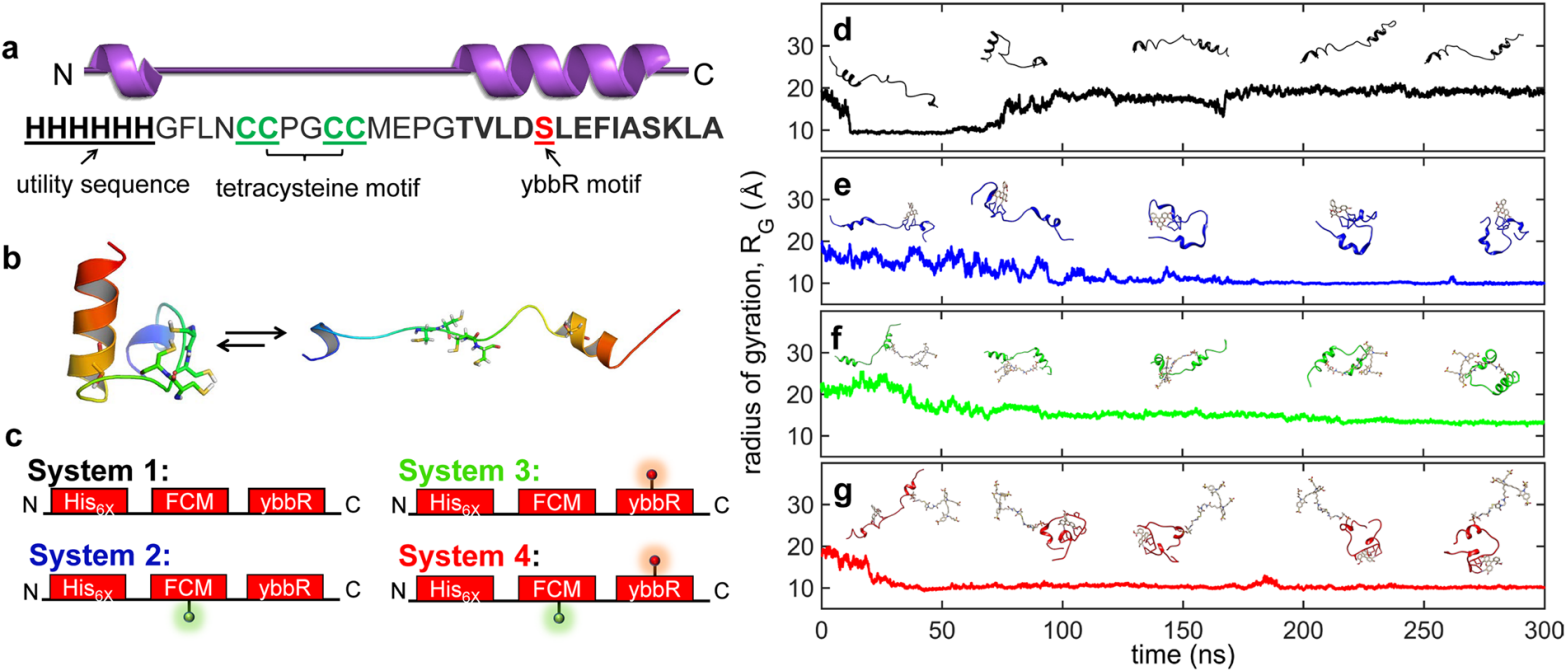
Molecular dynamics modelling of the FCM-ybbR peptide. (**a**) Secondary structure and primary sequence of the FCMybbR peptide design. The motifs of interest are underlined and color coded. (**b**) Modelled structure of FCMybbR obtained using PEP-FOLD and the linear structure obtained at the end of ASMD simulation. (**c**) Four systems were considered for MD simulations, System 1—with no fluorophores attached, System 2—with FlAsH attached, System 3—with CoA-AF647 attached, and System 4—with both fluorophores attached (Supplementary Movies S1–4). Colored beacons represent FlAsH (green) and CoA-AF647 (red). (**d–g**) Computed radius of gyration (R_G_) from 300 ns simulations for (**d**) FCMybbR, (**e**) FlAsH-FCMybbR, (**f**) FCMybbR-Co-AF647, and (**g**) FlAsH-FCMybbR-CoA-Alexa647. Conformational snapshots of the peptide in (**d–g**) correspond to those observed after 0, 75, 150, 225, and 300 ns, respectively.

For this study, we designed a synthetic 34-residue peptide containing two different site-specific labelling motifs (FCMybbR, Fig. 1a). The peptide has a hexahistidine (His_6x_) tag at the N-terminus, followed by the FCM and the ybbR motifs, respectively, both separated by a glycine spacer. The His_6x_ tag serves as a sequence for immobilization onto coverslips coated with His_6x_-specific antibodies^40^. The glycine spacers ensured that all four cysteines in the FCM motif (green letters) and the active serine site in the ybbR motif (red letter) are solvent accessible for efficient labelling (Fig. 1a).

### Molecular Dynamics

Structure-based modelling was performed to reveal the structural characteristics of FCMybbR. PEP-FOLD was used to model the 3D-folded structure of the peptide^41^ (Fig. 1a,b). A linear peptide model with no super-secondary interaction was generated by extending the PEP-FOLD model (Fig. 1b) with Adaptive Steered Molecular Dynamics (ASMD). The well-equilibrated stable linear structure with accessible cysteine and serine residues at the end of this simulation, was used as the starting structure for the subsequent simulation of four systems (Fig. 1c): FCMybbR with no fluorophores (System 1), with either FlAsH or CoA-AF647 fluorophore attached (System 2 and 3, respectively), and with both fluorophores attached (System 4).

**Figure 2:**
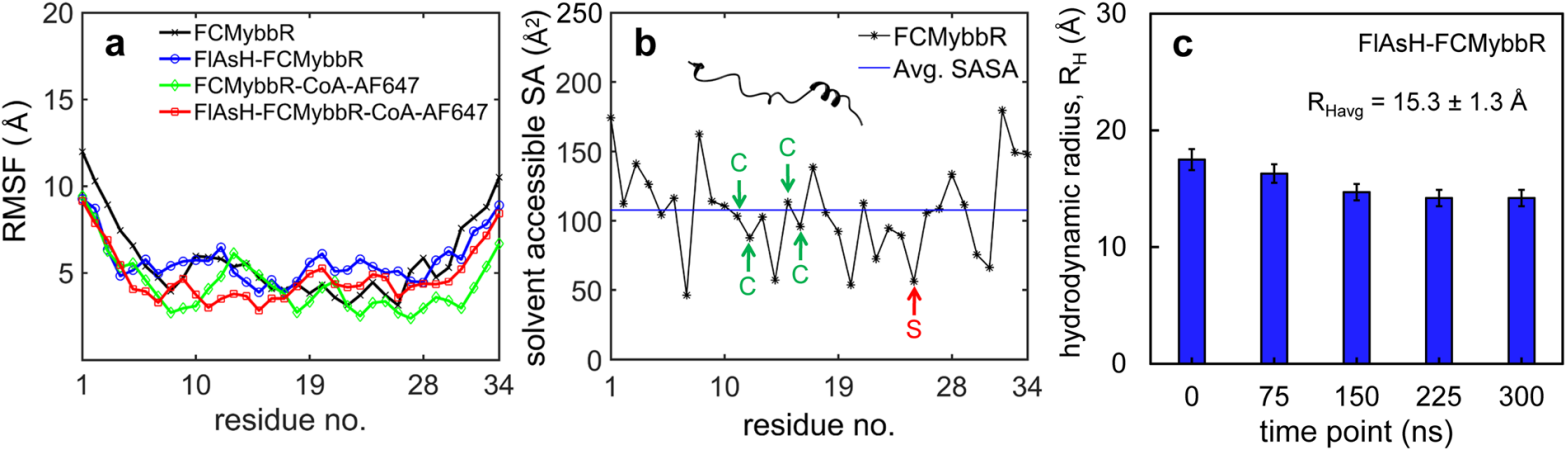
Structural stability, size and solvent accessibility of FCMybbR. (**a**) Root Mean Square Fluctuation (RMSF) of the alpha-carbons for FCMybbR as a function of residue number. (**b**) Residue-wise computation of the Solvent-Accessible Surface Area (SASA) of the FCM and ybbR motifs within the peptide. (**c**) The hydrodynamic radius of FlAsH-FCMybbR estimated using HYDROPRO from molecular dynamics snapshots at t = 0, 75 ns, 150 ns, 225 ns, and 300 ns.

The folding dynamics and conformational sampling of the peptide in each labelling state was studied using long molecular dynamic simulation trajectories (300 ns, Supplementary Movies S1–S4). The Radius of Gyration (R_G_) was calculated over the course of the simulations, and snapshots of specific peptide conformations for each case were obtained at 0, 75, 150, 225, and 300 ns (Fig. 1d–g). By the end of the MD trajectories, FCMybbR, FlAsH-FCMybbR, FCMybbR-CoA-AF647, and FlAsH-FCMybbR-CoA-AF647 converged to R_G_ values of 18.7, 10.0, 13.3, and 10.2 Å, respectively. Heatmaps depicting the time evolution of the predicted tertiary structure for each system were also generated and can be found in the Supplementary Information (Supplementary Fig. S1). To determine whether the peptide design would be conducive to labelling by relatively bulky fluorophores, the Root Mean Square Fluctuation (RMSF) and the solvent accessible surface area (SASA) for FCMybbR were calculated as a function of residue number. RMSF values were ~10 Å for the terminal residues and remained between 4 and 6 Å for the rest of peptide sequence (Fig. 2a). SASA values varied between 46.2 and 179.5 Å^2^, with an average of 107.7Å^2^ (Fig. 2b). Using previously published methodology^42^, the relative accessible surface area compared to the maximum possible accessible surface area for the cysteines within the FCM-motif and the active serine within the ybbR-motif were calculated to be 67% and 39%, respectively. This suggests that FCMybbR is a good candidate for dual labelling with FlAsH-EDT_2_ and CoA-AF647, as both motifs remain largely solvent-accessible. Using HYDROPRO^43^, the hydrodynamic radius (R_H_) of FlAsH-FCMybbR was computed using the generated PDB files at 0, 75, 150, 225, and 300 ns (Fig. 2c). The average R_H_ found was 15.3 ± 1.3 Å, a value which is consistent with the value derived from FCS measurements (*vide infra*).

### Labelling with FlAsH-EDT_2_ and CoA-AF647

**Figure 3:**
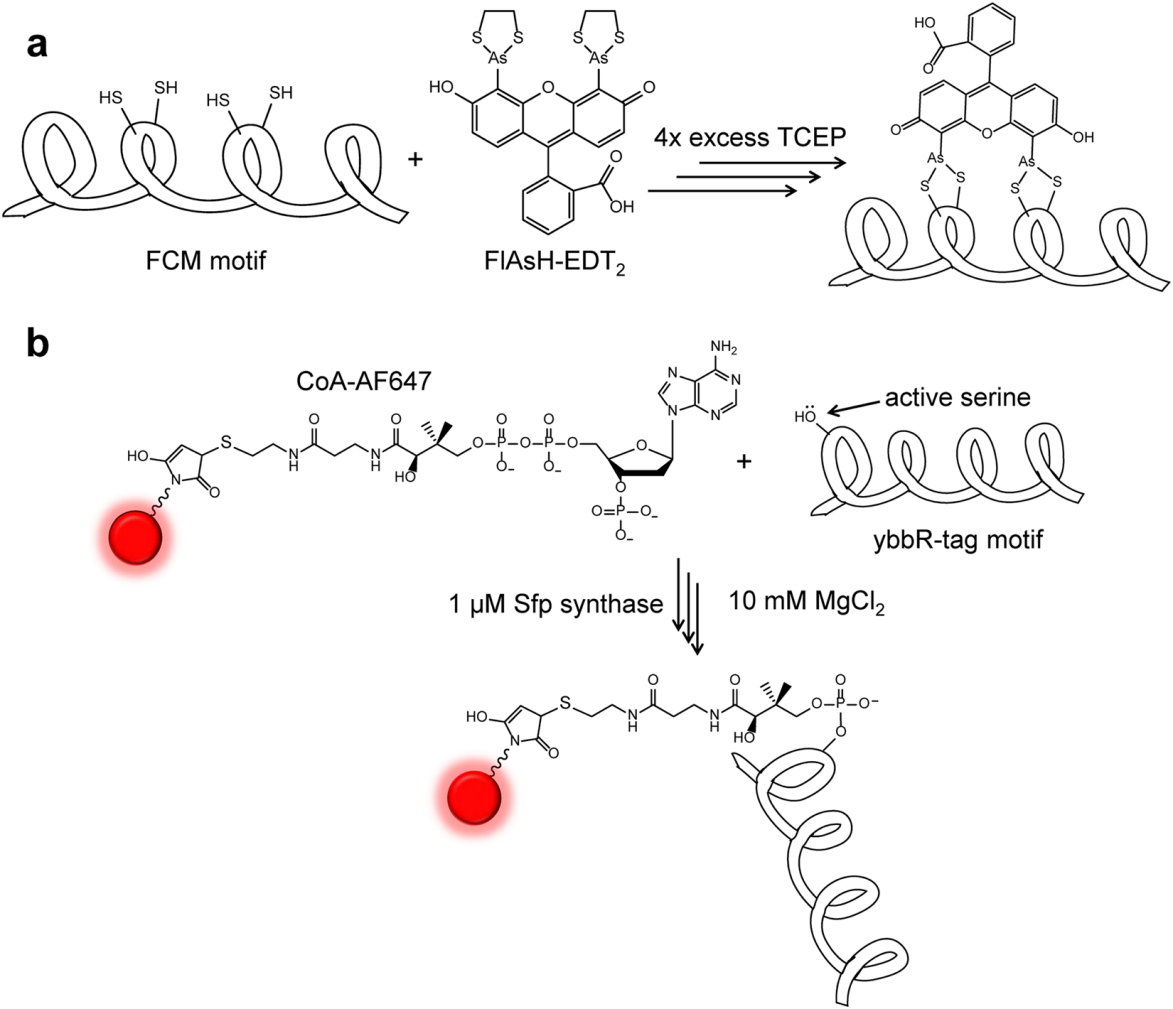
Labelling the FCM motif with FlAsH and the ybbR motif with CoA-AF647. (**a**) Labelling scheme and mechanism of attaching FlAsH-EDT_2_ to the FCM motif in the presence of the reducing agent TCEP. (**b**) Labelling scheme and mechanism for attaching CoA-AF647 to the ybbR motif in the presence of MgCl_2_ and the enzyme Sfp synthase.

The procedure used here for FlAsH labelling is outlined in Fig. 3a. The optimal labelling stoichiometry between FlAsH-EDT_2_ and FCMybbR was determined prior to purification, by fixing the concentration of FlAsH-EDT_2_ and varying the FCMybbR:FlAsH-EDT_2_ ratio. The efficiency of labelling was characterized in solutions buffered at pH 7.4 (phosphate buffered saline, PBS, 150 mM NaCl) using FCS. The optimal labelling reaction time was determined by adding 100 μM FCMybbR to a solution containing 1 μM FlAsH-EDT_2_ and 4 mM tris-2-carboxyethyl-phosphine (TCEP) at room temperature in the dark. Previously published protocols indicated that at least 10x excess of protein compared to FlAsH-EDT_2_ is required for labelling^44^. Based on the gradual shift observed in the FCS curves to longer decay times (larger R_H_ values), the FlAsH labelling reaction was completed within 3 hours after incubation.

To label the FCMybbR motif with AF647, the fluorophore was first conjugated to Coenzyme A (CoA) via maleimide-thiol coupling (Fig. 3b). The purifications of FlAsH-FCM-ybbR and of CoA-AF647 were performed using Anion Exchange Chromatography (AEC) based on the net charge of each species (see Methods, Supplementary Fig. S2). Covalent attachment of CoA-AF647 to ybbR was achieved via incubation with 1 μM Sfp synthase and 10 mM MgCl_2_ in PBS buffer (150 mM NaCl, pH 7.4, 2 mM TCEP) for 5 hours at room temperature (see Methods).

### Spectra, Lifetimes and Anisotropy Decays of FlAsH-FCM-ybbR

**Figure 4:**
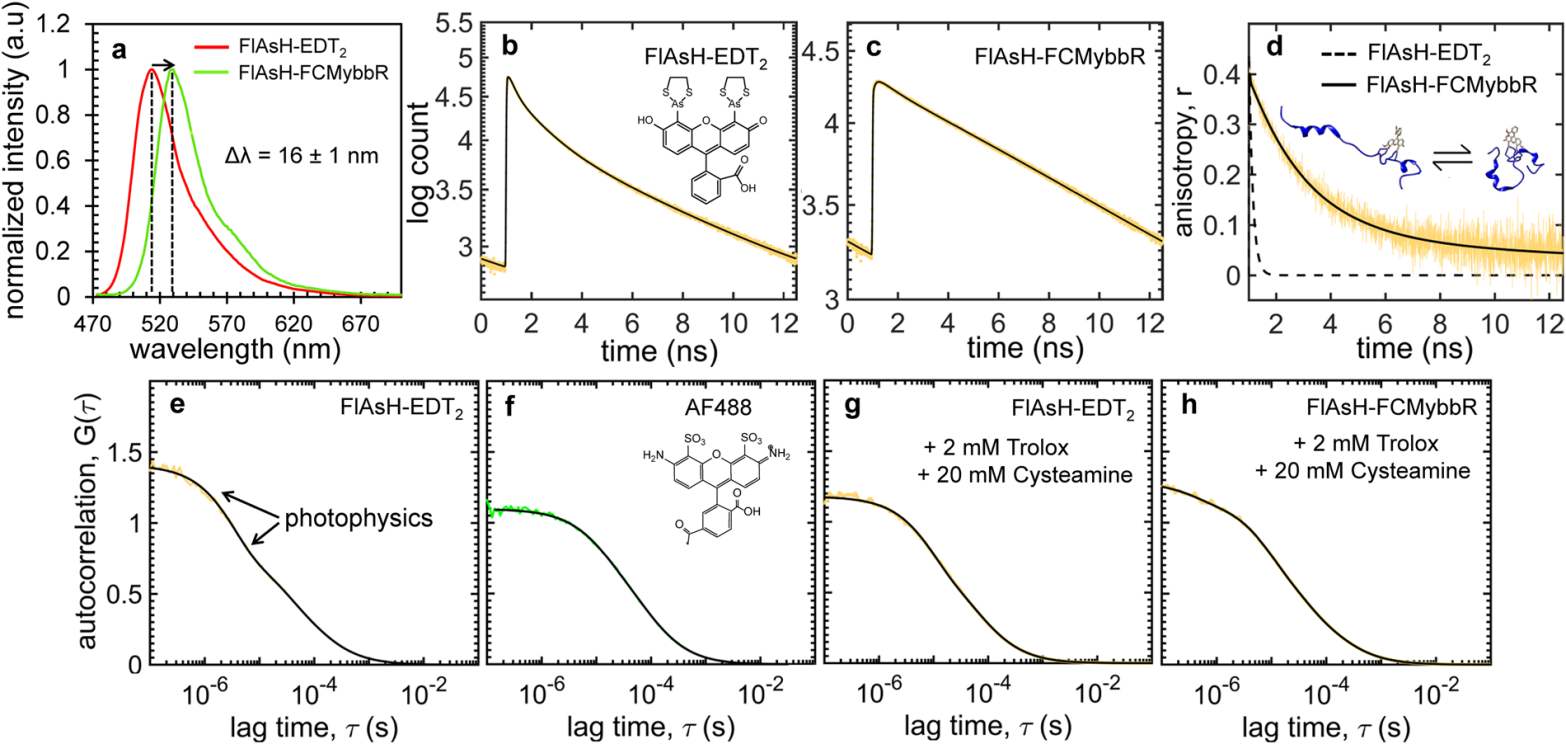
Spectroscopic characterization of FlAsH. (**a**) Emission spectra of free FlAsH-EDT_2_ (red) and purified FlAsH-FCMybbR (green); the vertical dashed lines and arrow illustrate the 16 ± 1 nm redshift of the emission peak upon labelling. (**b**) Fluorescence decay data of free FlAsH-EDT_2_ and (**c**) FlAsH-FCMybbR. (**d**) Fluorescence anisotropy decay of free FlAsH-EDT_2_ (dashed line) compared to FlAsH-FCMybbR (yellow). Solid black lines in (**b–d**) are fitted exponential decays according to equations (1,3). FCS data for (**e**) FlAsH-EDT_2_ (yellow), (**f**) AF488 (green), and (**g**) FlAsH-EDT_2_ in the presence of 20 mM cysteamine, 2 mM Trolox (yellow). (**h**) purified FlAsH-FCMybbR in the same conditions as (**g**). All FCS curves were normalized at 10^−6^ s for comparison purposes. Solid lines in (**e–h**) are the fitted FCS curves according to equation (4). The lifetime, anisotropy decay and FCS fitting parameters are summarized in Supplementary Tables S1 and S2.

After AEC purification, the fluorescence properties of FlAsH-FCMybbR were measured using fluorimetry and time-resolved fluorescence decay (tr-FD) and were compared to free FlAsH-EDT_2_ (Fig. 4). The emission spectra of ~10 μM free FlAsH-EDT_2_ was acquired and compared to ~1 μM of purified FlAsH-FCMybbR in PBS buffer (Fig. 4a). Despite the fluorogenic properties of FlAsH-EDT_2_, residual fluorescence was still detected, possibly arising from small amounts of fluorescein impurities, with a major peak centered at 514 ± 1 nm (Fig. 4a, red).When bound to the peptide, FlAsH exhibited a red-shift of 16 ± 1 nm, with a maximum at 530 ± 1 nm (Fig. 4a, green), which is in agreement with previous reports^44^. Further, the absence of a discernable emission peak at 514 nm and the appearance of the peak at 530 nm are indicative of successful labelling and purification of FlAsH-FCMybbR.

Time-resolved polarized detection upon pulsed excitation was done using a custom-built confocal microscope with time-correlated single-photon counting^45^. The samples measured were 10 μM FlAsH-EDT_2_ and 1 μM FlAsH-FCMybbR (Fig. 4b–d). The reconstructed isotropic decay curve for FlAsH-EDT_2_ (Fig. 4b) was best fit to a sum of three exponential terms (equation (1)) with lifetimes of τ_L1_ = 0.24 ns, τ_L2_ = 1.13 ns, and τ_L3_ = 4.2 ns, respectively. The fractions of the two shorter lifetimes (τ_L1_, τ_L2_) are much larger than the fraction of the longest lifetime (τ_L3_): *B*_1_ = 54% and *B*_2_ = 35%, vs. *B*_3_ = 11%, respectively (Supplementary Table S2). The two shorter lifetimes most likely correspond to rapid transitions between the fluorescent and quenched state of FlAsH-EDT_2_, which may be caused by fast rotational fluctuations of the Arsenic-aryl (As-aryl) bond of FlAsH-EDT_2_. These findings are in agreement with the rotamer-restricted fluorogenicity model, which was empirically evaluated on ReAsH-EDT_2_ by increasing the viscosity of the measured solution^17^. This model describes quenching by PET between EDT_2_ and the fluorophore’s conjugate π-bond system^13,17^ and implies that the more rotational freedom the As-aryl bonds experience, the more efficient the quenching becomes. Thus, the slowest decay could arise from a specific set of rotameric states of the As-aryl bond, in which the rotational freedom of the bond is restricted, or, alternatively, from fluorescein impurities, *vide infra*.

The fluorescence decay curve measured for FlAsH-FCMybbR was best fit to two exponential terms, with lifetimes of τ_L1_ = 0.55 ns and τ_L2_ = 4.72 ns, and amplitudes of *B*_1_ = 17% and *B*_2_ = 83%, respectively (Fig. 4c, Supplementary Table S2). The longer lifetime, τ_L2_, is similar to the published fluorescence lifetime of FlAsH bound to the FCM motif, i.e., 4–5 ns^46,47^. Thus, the significant increase of the long-lifetime fraction compared to the free dye is indicative of successful attachment of FlAsH to the target peptide. The presence of a minor short-lifetime fraction could be due to the free dye, although the anisotropy decay and the FCS data do not support this, *vide infra*. Alternatively, some FCMybbR conformations could favor PET between FlAsH and histidines, as indicated by MD simulations showing that the N-terminal histidines come within close proximity (~5 Å) of the fluorophore (Supplementary Fig. S3, Supplementary Movie S2).

Rotational correlation times of the free and the peptide-bound FlAsH were estimated by fitting the anisotropy decay curves to equation (3) (Fig. 4d). The curve for the free dye was best fit to a single exponential decay with a lifetime of ρ_1_ = 0.14 ns, whereas the curve for FlAsH-FCMybbR was best fit to an exponential term with ρ_1_ = 2.29 ns and an offset term (ρ_2_ → ∞), with amplitudes of *A*_1_ = 86% and *A*_2_ = 14%, respectively (Supplementary Table S2). The absence of a fast anisotropy decay on the order of 0.1–0.2 ns suggests that the purified labelled peptide sample did not contain free FlAsH-EDT_2_ or fluorescein impurities. Further, it supports the conclusion that the short fluorescence lifetime (~0.55 ns) is caused by PET quenching, *vide supra*. The hydrodynamic radius estimated from the rotational correlation lifetime of FCMybbR is ~13 Å, which is agreement with the value obtained from MD simulations using HYDRPRO (~15 Å, Fig. 2c) and the value measured by FCS, *vide infra*. The offset term accounts for slower rotational dynamics that may arise due to the existence of extended conformations of the peptide (Fig. 1e, Supplementary Fig. S3b). MD simulations show that transitions between peptide conformational states are slow compared to the fluorescence lifetime (Fig. 1e), which can lead to incomplete decay of the anisotropy within the time window between successive excitation pulses (See Methods).

### Photophysical characterization of FlAsH-EDT_2_ and FlAsH-FCMybbR

FCS data acquired on 500 nM FlAsH-EDT_2_ were normalised at 10^−6^ s and fitted to equations (4,5), to obtain the hydrodynamic size (R_H_) and the photophysics (triplet lifetimes) of the free FlAsH fluorophore. The results were compared with those obtained for AF488, a standard blue dye used for single-molecule studies (Fig. 4e–h, Supplementary Table S2). The FlAsH correlation curve was best fit to a single-diffusion component (τ_D = _81 ± 1 μs), corresponding to an R_H_ value of 9.1 ± 0.2 Å, and two triplet-lifetime decay constants, t_1_ = 3.4 ± 0.1 μs and t_2_ = 28 ± 3 μs (Fig. 4e, Supplementary Table S2). The effective concentration of fluorescent species was calculated to be ~2 nM, which is most likely due the presence of fluorescein impurities or a small fraction of FlAsH-EDT_2_ in a non-quenched state, *vide supra*. Using the ratio between the FCS-calculated concentration and the concentration assuming 100% non-fluorescent FlAsH-EDT_2_ content, the corresponding percentage of species in the fluorescence state was ~0.4%. This estimated value is similar to previously published results (0.05–0.5%)^44^. Supplementation with excess 1,2-Ethanedithiol (EDT) has been shown to suppress fluorescence from these states of the fluorphore^20^.

The FCS correlation curve for AF488 was best fit to a single-diffusion component (τ_D = _67 ± 1 μs), corresponding to a R_H_ value of 6.3 ± 0.3 Å, and a single triplet-lifetime decay constant, t_1_ = 11 ± 1 μs (Fig. 4f). The hydrodynamic radius of FlAsH-EDT_2_ is larger than the measured value for AF488 despite having similar chemical structure. This discrepancy could be caused by the presence of long-lived triplet states (arrows in Fig. 4e), which are absent from the FCS curve for AF488. To test this hypothesis, FCS measurements on FlAsH-EDT_2_ were repeated in the presence of 2 mM Trolox and 20 mM cysteamine (Fig. 4g). These are known photo-protectant agents for enhancing brightness and reducing triplet-state and singlet-oxygen transitions (photo-blinking) of Alexa fluorophores^48^. In the presence of these reagents, only one triplet-lifetime decay constant was observed, 9 ± 1 μs, and the overall triplet population was reduced by ~50% (Supplementary Table S2). Most remarkably, the diffusion time and the calculated hydrodynamic radius, *i.e*., τ_D_ = 58 ± 1 μs, and R_H_ = 6.6 ± 0.3 Å, were closer to the value predicted by HYDROPRO (R_H_ = 6.9 ± 0.1 Å).

For FlAsH-FCMybbR in the presence of quenching agents (Fig. 4h) the correlation decay curve was best fit to a single-component diffusion model (τ_D_ = 108 ± 1 μs) with three triplet lifetimes (t_1_ = 0.37 ± 0.07 μs, t_2_ = 8 ± 1 μs, and t_3_ = 36 ± 2 μs). The R_H_ for FlAsH-FCMybbR was estimated to be 12.4 ± 1.2 Å, which is almost twice as high as that of FlAsH-EDT_2_ (Supplementary Table S2). This is also reasonably close to the HYDROPRO value calculated from the MD snapshots (R_Havg_ = 15.3 ± 1.3 Å, Fig. 2c). The emergence of the fast and slow triplet components (t_1_ and t_3_) with an additional triplet population of ~40%, is most likely due to PET by the N-terminal histidines, as indicated by MD simulations (Supplementary Fig. S3, Supplementary Movie S2).

### Single-molecule TIRF imaging of *in-situ* orthogonal labelling

**Figure 5:**
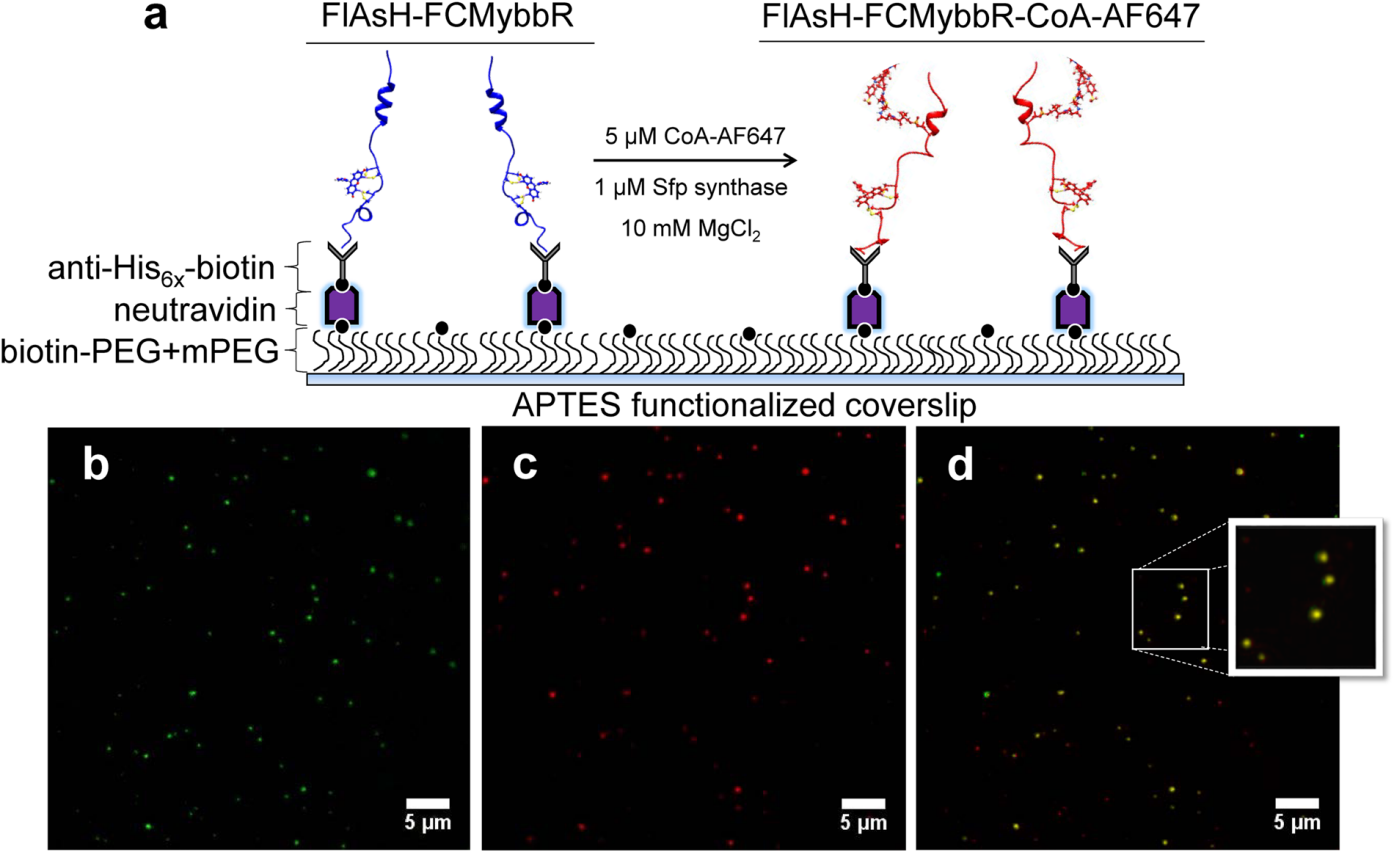
TIRF imaging of peptide labelling with FlAsH and AF647. (**a**) Immobilization scheme for anchoring FlAsH-FCMybbR and *in-situ* labelling with CoA-AF647. (**b**) TIRF image of FlAsH-FCMybbR immobilized on a coverslip coated with anti-His_6x_ upon excitation at 473 nm (green spots). (**c**) TIRF image of CoA-AF647 when bound to FlAsH-FCMybbR after incubation with 1 μM Sfp and 10 mM MgCl_2_ upon illumination with 633 nm light (red spots). (**d**) TIRF image overlay displaying co-localization of the fluorescence signal of FlAsH and CoA-AF647 when both are bound to FCMybbR (yellow spots).

TIRF microscopy was used to explore whether individual FlAsH-FCMybbR molecules can be detected by fluorescence and to assess the efficiency of *in-situ* labelling of CoA-AF647 onto surface-anchored FlAsH-labelled peptides (Fig. 5). FlAsH-FCM-ybbR was first immobilized via anti-His_6x_ antibodies onto the bottom coverslip of a custom-made flow chamber, as described previously^21^. The fluorescence of single peptide-bound FlAsH molecules was bright enough to be detected by TIRF (Fig. 5a). After washing off the excess FlAsH-FCMybbR that did not immobilize, a solution of 1 μM of Sfp synthase, 10 mM MgCl_2_ and 5 μM CoA-AF647 was incubated in the chamber for 2 hours, followed by thorough washing with the buffer solution.

TIRF data acquired after the incubation and washing steps show the appearance of single AF647 spots on the surface (Fig. 5b). TIRF images of FlAsH and CoA-AF647 fluorescence from the same area were pseudo-coloured and overlaid using ImageJ^49^. On average, 60–80% of the spots in the two channels overlapped across 15 different 50 μM × 50 μM regions of interests (yellow spots, Fig. 5d). The lower bound of observed co-localization (~60%) may indicate that more extended, solvent exposed conformations of FCMybbR are easier for CoA-AF647 to access, whereas more compact conformations may hinder labelling.

### Multiparameter single-molecule fluorescence analysis of FlAsH-FCM-ybbR-CoAF647

**Figure 6:**
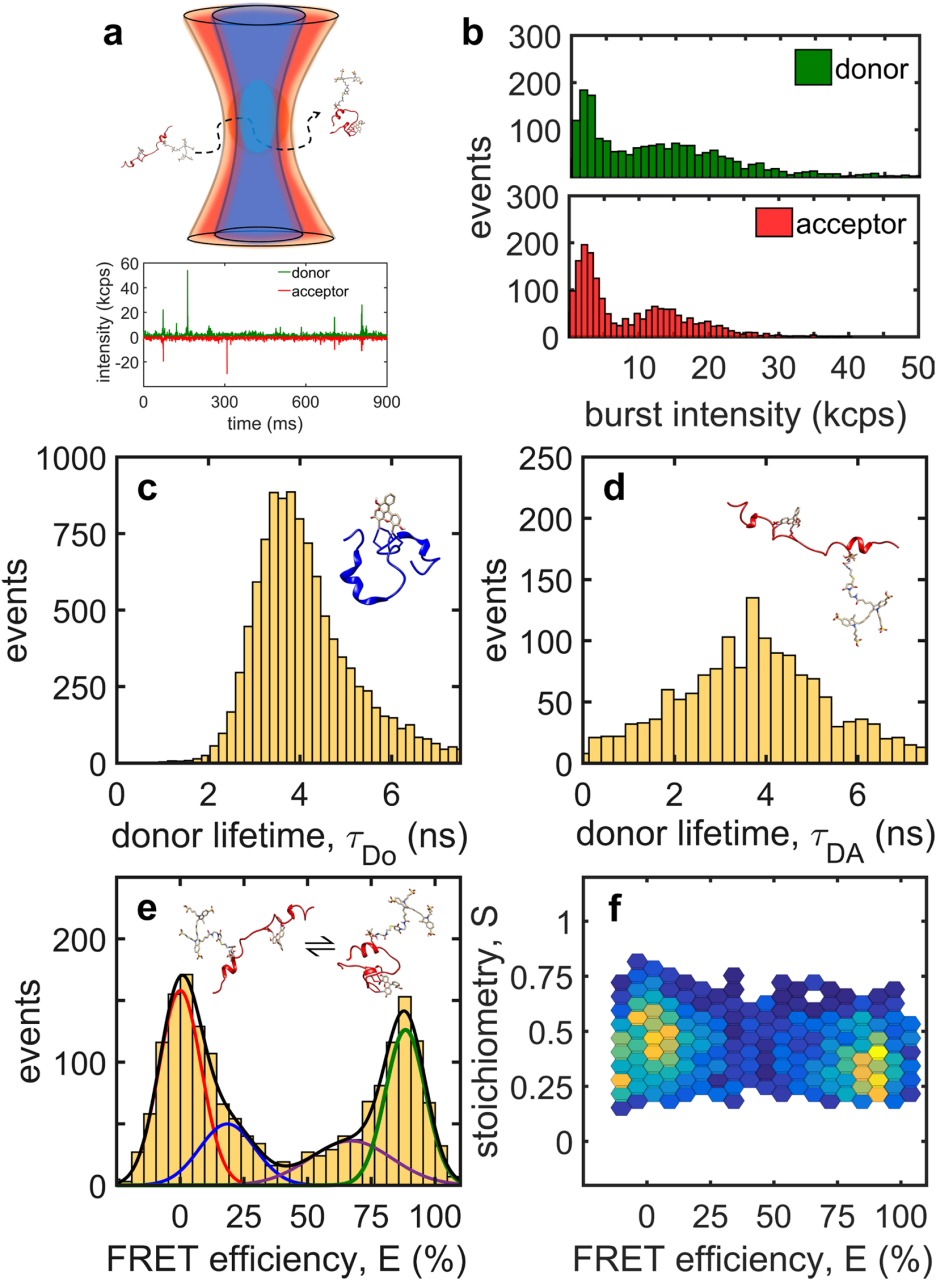
Single-molecule burst analysis of FlAsH-FCMybbR-CoA-AF647. (**a**) Schematic depiction of single FlAsH-FCMybbR-CoA-AF647 diffusing through the confocal detection volume illuminated with blue and red light. (**b**) Burst brightness distribution of the donor (FlAsH) and acceptor (CoA-AF647) for dually-labelled peptides upon 480 nm excitation. (**c**,**d**) The distribution of MLE donor lifetimes in the absence of the acceptor (τ_Do_), and in the presence of the acceptor (τ_DA_). (**e**) FRET histogram calculated from intensity bursts of FlAsH-FCMybbR-CoA-Alexa647 using equations (6,7) and fitted to a sum of Gaussian components with major peaks at *E* = 0% (red) and *E* = 90% (green) with side peaks at *E* = 19% (blue) and *E* = 67% (purple) which most likely describe bridging effects between the two major FRET peaks. (**f**) 2D plot of donor-acceptor stoichiometry calculated using equation (8) as a function of FRET efficiency. The measurements were performed in the presence of 20 mM cysteamine and 0.01% Tween20 in a PBS buffer (0 M NaCl, pH 7.0).

A multiparameter fluorescence (MPF) confocal microscope^50^ with alternating-laser excitation (ALEX)^39^ was used to characterize the dually labelled peptide, FlAsH-FCMybbR-CoA-AF647, at the level of single- molecule bursts of fluorescence (Fig. 6a). The experiments were performed in the presence of 20 mM cysteamine, and 0.01% Tween20 in PBS buffer (pH 7.0) with 0 M NaCl to provide similar conditions to those used for MD simulations^41^. Addition of 0.01% Tween20 prevents non-specific adsorption of peptides to coverslips and related FRET artefacts^51^. ALEX interleaves the “normal” donor-selective excitation with direct excitation of the acceptor and allows the categorization of fluorescence intensity bursts as donor-only, acceptor-only, or smFRET bursts.

MPF data analysis provided information pertaining to the molecular brightness of the donor and the acceptor (Fig. 6b), the distribution of donor fluorescence lifetime in the absence and in the presence of the acceptor (Fig. 6c,d), and the FRET efficiency distribution (Fig. 6e). Single-molecule bursts from peptide-bound FlAsH exhibited a bimodal distribution of intensities, with peaks at ~3 kcps and ~15 kcps (Fig. 6b). The average background level was ~0.5 kcps. The distribution of burst intensities from peptide-bound AF647 was also bimodal and in a similar range, between 0 and 40 kcps. The occurrence of two molecular brightness populations for both the donor and the acceptor points to two FRET populations. The distribution of fluorescence lifetimes from donor-only bursts shows a single population with an average lifetime of τ_Do_ = 4.37 ± 0.80 ns (Fig. 6c). This value is very close to the major component detected by ensemble lifetime measurements, 4.72 ns (Fig. 4c, Supplementary Table S1); the minor fast component (0.55 ns) was not detected at the burst level, most likely due to filtering out the weak bursts in the data analysis. In the presence of the acceptor, the distribution of donor lifetimes broadened significantly towards shorter values ( < 2 ns), which is consistent with quenching of donor fluorescence by singlet energy transfer to the acceptor (Fig. 6d).

The FRET efficiency, *E*, was calculated for each burst using equations (6,7) and the values obtained were used to build a smFRET histogram (Fig. 6e). The smFRET distribution for the FCMybbR peptide shows two major peaks around *E*_1_ = 0% (red) and *E*_2_ = 90% (green), with asymmetric tails towards higher and lower FRET values, respectively. The donor-acceptor stoichiometry, *S*, for each of these FRET populations was calculated using equation (8) and varied between 0.2 and 0.8 (Fig. 6f). This indicates that the measured zero-FRET peak originates from peptides with both the donor and the acceptor, not from peptides labelled only with the donor.

**Figure 7:**
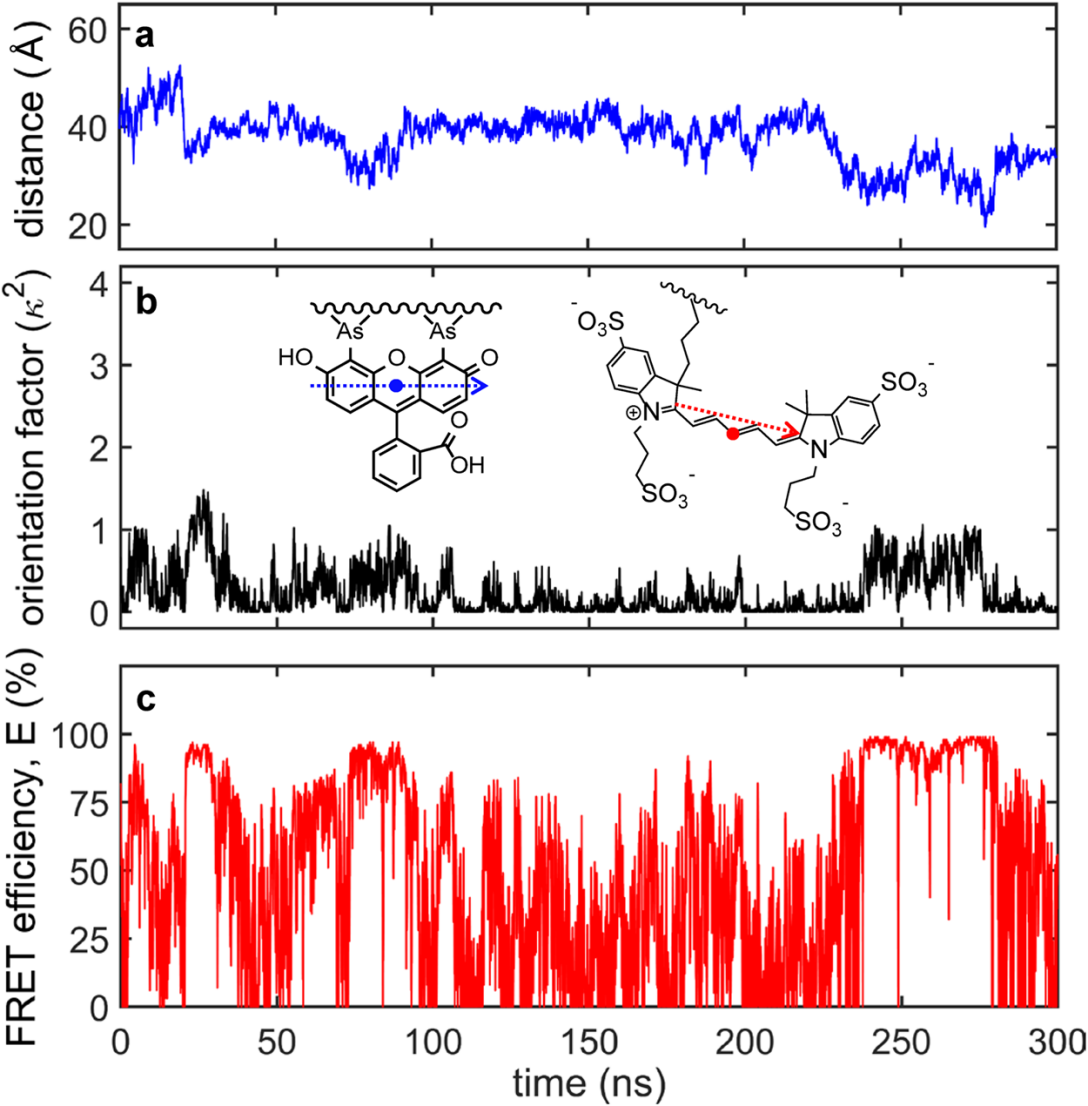
FRET calculations based on molecular dynamics parameters. (**a**) MD-estimated centre-to-centre distance fluctuations between the FlAsH and AF647 labels bound to FCMybbR. (**b**) Computed orientation factor, κ^2^_calc_, between the transition dipoles of FlAsH (blue arrow), and AF647 (red arrow) using equation (9). (**c**) Computed FRET efficiency over the span of 300 ns using the time-correspondent κ^2^_calc._ from (**b**) and the distance from (**a**).

To interpret the experimental FRET data, the distance, *R*, and the orientation factor between the donor and acceptor transition dipoles, κ^2^ (see equation (9)), were computed in the MD simulations (Fig. 7a–b, Supplementary Movie S4). Using these MD parameters, the “instantaneous” FRET efficiency was calculated at each time-point over the course of the 300-ns MD simulation using the Förster equation^52^ (Fig. 7c). The donor-acceptor distance varied between 20 and 50 Å, with an average of ~40 Å (Fig. 7a, Supplementary Fig. S5a), whereas κ^2^ varied between 0 and 1.5, with rather discrete jumps between 0 and ~1.0 (Fig. 7 b). The restricted range for κ^2^ compared to the theoretical range between 0 and 4 is due to FlAsH being rigidly attached to the FCM motif of the peptide (Supplementary Fig. S4). This is consistent with the absence of a fast sub-ns depolarization component in the anisotropy decay data (Fig. 4d).

The κ^2^ and FRET trajectories shows that the two quantities are strongly correlated (Fig. 7b,c, Supplementary Fig. S5b,d), whereby the “instantaneous” efficiencies span the entire theoretical range, with a clear high-FRET state at *E*≈100% and a low-FRET population with *E* < 20% (Fig. 7c, Supplementary Fig. S5d). The isotropic average value ${\langle {\kappa }^{2}\rangle }_{iso}$ = 2/3 is typically used in the Förster equation^52^, which yields a Förster radius (*R*_0_) of 52 Å for the FlAsH-AF647 pair. With this *R*_0_ and the MD-simulated donor-acceptor distances (Fig. 7a), the calculated FRET efficiency was found to vary only between 50% and 100% (Supplementary Fig. S5c). Conversely, using the time-averaged κ^2^ over the course of the 300-ns MD simulation, ${\langle {\kappa }^{2}\rangle }_{MDavg}$ = 0.245, the calculated *R*_0_ was 43 Å and yielded a range in FRET efficiencies between 20% and 100% for the peptide (Supplementary Fig. S5c). This suggests that the observed efficiencies above 20% in the smFRET measurements are consistent with fast conformational switching of the peptide as revealed by MD (Fig. 1g), whereas the values below 20% are consistent with interactions between dyes and the peptide that lead to deviations from the isotropic dipole orientation assumption (Fig. 7a–c, Supplementary Fig. S5b–d and Movie S4). This is perhaps expected given the chemistry of FlAsH labelling via the tetracysteine motif, but it warns about simplistic distance inferences when using it for smFRET measurements.

Whereas MD simulations are capable of identifying the expected spread of FRET efficiencies within a (sub)microsecond time-window, the relative populations between these states does not match to the measured smFRET distribution, which represents a time-averaged distribution over 0.1–1 ms, *i.e*., the duration of the burst. The relative populations for the low-, and high-FRET species will not only depend on the stability of the conformation but also the relative brightness of the donor and acceptor in each state. In the case of FCMybbR, alternative quenching pathways exist, implying that the brightness of FlAsH and its capability to transfer energy to AF647 might be modulated by the N-terminal His_6x_-tag (Fig. 4g, Supplementary Fig. S3 and Movie S4). Therefore, the selection criteria for the labelling site for FlAsH when performing smFRET may be quite different than when using it as a PET probe for fast conformational changes.

## Conclusions

The arsenical hairpin derivative of fluorescein, FlAsH, offers a highly specific labelling strategy of proteins via a small tetracysteine motif (FCM) which can be inserted in solvent accessible regions, typically looped or disordered regions. As such, FlAsH has been used for fluorescence studies of membrane receptors in purified samples and in live cells^19–21^. Its small size makes it an attractive alternative to GFP which is bulky and, while versatile for imaging in live cells, is not an ideal fluorophore for single-molecule spectroscopy. Here, we demonstrated the suitability of FlAsH as a single-molecule fluorophore for a sleuth of single-molecule techniques using a minimal peptide (FCMybbR) designed for orthogonal labelling with FlAsH and a red dye, AF647. As a fluorogenic probe, FlAsH exhibits a significant increase in brightness upon labelling, accompanied by a 16 nm red-shift of the emission spectrum. The analysis of fluorescence intensity fluctuations for the peptide-bound fluorophore revealed the presence, apart from the intrinsic triplet-state blinking, of two other photoblinking processes with lifetimes of ~0.4 μs and slow ~35 μs. This reflects the dynamic quenching of the FlAsH via photoinduced electron transfer by the N-terminal histidines in FCMybbR, which were shown to come in close contact with the fluorophore in molecular dynamics simulations. This sensitivity of FlAsH to PET by aromatic amino-acids could be used to measure the conformational dynamics of proteins by FCS, as shown for other fluorophores^25,29^.

We performed orthogonal *in situ* labelling of FlAsH and CoA-AF647 onto FCMybbR, as demonstrated by co-localization TIRF microscopy. This dual labelling scheme “locks” each fluorophore at specific sites, which is advantageous over standard thiol-reactive dyes, whereby the fluorophores can attach at either site and thus complicate the data interpretation. TIRF imaging also demonstrated that the FlAsH probe is bright enough for single-molecule detection. In addition, using the dual-labelled test peptide, we detected single-molecule fluorescence bursts and measured energy transfer between FlAsH and AF647. The burst intensity analysis showed a molecular brightness distribution for FlAsH comparable to AF647, which is a bright organic dye typically used in single-molecule studies. Fluorescence lifetime and smFRET histogram analysis further confirmed the suitability of FlAsH as a donor in smFRET studies when paired with red dyes such as AF647. Rather unexpectedly, a low-FRET population was observed, which might originate not from a large donor-acceptor distance but rather from unfavorable orientations between the two dyes, as shown in molecular dynamics simulations. The smFRET data obtained from this dye-pair is encouraging for future studies, and provides a solid framework for accurate interpretation of smFRET distributions when accompanied by molecular dynamics simulations. The FlAsH-FCM and ybbR-CoA-AF647 labelling tandem seems to be a promising replacement for bulky and photo-physically unstable fluorescent proteins, especially for smFRET and single-molecule tracking studies of membrane receptors in live cells.

## Methods

### Labelling of FCM-ybbR with FlAsH and CoA with AF647

To prevent the formation of disulfide bonds, 300 μM of FCM-ybbR (GenScript, USA) was incubated with 2 mM tris-2-carboxyethyl-phosphine (TCEP, Sigma Aldrich, cat. No. C4706) in a solution buffered at pH 7.0 (phosphate buffered saline, PBS, 0 M NaCl) and flushed with argon gas for 15 minutes. FlAsH-EDT_2_ was then added to a concentration of 27 μM, which resulted in 10x molar excess of FCM-ybbR (270 μM). This solution was gently stirred and incubated in the dark at room temperature for 3 hours. Prior to labelling with AF647-malemide, 250 μM of CoA (Sigma Aldrich, C3019) was incubated with 2 mM TCEP in PBS buffer (150 mM NaCl, pH 7.4) and flushed with argon. AF647-malemide was then added to a concentration of 500 μM and the solution was gently stirred and incubated in the dark at room temperature for 5 hours.

### Purification of FlAsH-FCM-ybbR and CoA-AF647

FlAsH-labelled FCM-ybbR and AF647-labelled CoA were purified via Anion Exchange Chromatography (AEC) using a HighQ strong anion exchange cartridge (Bio-Rad Laboratories Inc., cat. No. 7324120) coupled to a Low-pressure chromatography system capable of simultaneously monitoring changes in optical density and conductivity (Bio-rad Laboratories Inc., BioLogic LP). For optimal separation of FlAsH-FCMybbR, a salt gradient varying from 0 to 2 M NaCl in PBS buffer (pH 8.5) was used. The optimal pH was chosen using the theoretical net charge versus pH plot of FCMybbR generated using built-in MATLAB code (*i.e*., isoelectric), which predicted a net charge of −5 at pH 8.5 (Supplementary Fig. S2a). This position provided the greatest charge separation between free FlAsH-EDT_2_ (−2), unlabeled peptide (−5) and FlAsH-labeled peptide (−7), respectively (Supplementary Fig. S2b). CoA-AF647 purification was performed using the same salt gradient (0 to 2 M NaCl) in tris(hydroxymethyl)aminomethane (TRIS) buffer (pH 8.5), and provided optimal charge separation between free AF647 (−3), unlabeled CoA (−4), and labelled CoA-AF647 (−7), respectively (Supplementary Fig. S2c).

### Labelling and purification FlAsH-FCM-ybbR-CoA-AF647

The purified FlAsH-FCM-ybbR (~1 μM) was incubated with 1 μM Sfp synthase (New England BioLabs, cat. No. P9302S), 10 mM MgCl_2_, and 5 μM CoA-AF647, in PBS buffer (150 mM NaCl, pH 7.4) at room temperature with gentle stirring for 5 hours. Separation and concentration of FlAsH-FCM-ybbR-CoA-AF647 (MW~ 6.2 kDa) were performed using centrifugal filter units with a 3 kDa molecular weight cut-off (Sigma Aldrich, Amicon® Ultra centrifugal Filters, cat. no. UFC5003). Centrifugation was repeated four times with continual supplementation of PBS buffer until a final concentration of ~500 nM was achieved, which was stored at 4 °C.

### Fluorescence lifetime and anisotropy decay analysis

Polarization-resolved fluorescence decay curves were constructed by binning the arrival time of photons with orthogonal polarization. FlAsH fluorescence lifetimes were estimated by fitting the isotropic decay curve to a multi-exponential decay model:

1 $$F(t)=\frac{1}{\sum _{1}^{i}{B}_{i}}\cdot [\sum _{1}^{i}\frac{{B}_{i}}{1-{e}^{-T/{\tau }_{Li}}}\cdot {e}^{-t/{\tau }_{Li}}]$$

where *B*_*i*_ is the fraction of species with the lifetime *τ*_*Li*_ and *T* is the excitation pulse period (12.5 ns)^53^.

The fluorescence anisotropy is defined by the following ratio between parallel and perpendicular components of the collected fluorescence emission,

2 $$r(t)=\frac{{I}_{||}(t)-G{I}_{\perp }(t)}{{I}_{||}(t)+2G{I}_{\perp }(t)}$$

The anisotropy decay time constants, *i.e*., the rotational correlation times, were estimated by fitting the anisotropy decay curve to a multi-exponential model:

3 $$r(t)={r}_{o}[\sum _{1}^{i}{A}_{i}\cdot {e}^{-t/{\rho }_{i}}]$$

where *r*_o_ is the fundamental anisotropy of the dye (~0.4) and *A*_i_ is the total fraction of species with rotational correlation time *ρ*_*i*_^54^. In order to account for repetitive excitation in our setup, we summed the fluorescence decay curves over 5 preceding pulses as described previously^53^. The uncertainty in the fitted parameters was estimated using statistical bootstrapping^55^. Goodness of the fit and validation of the number of free parameters were assessed through minimization of χ^2^ and the Akaike information criterion (AIC), respectively^56^.

### Fluorescence Correlation Spectroscopy

To characterize the hydrodynamic radius and the photophysical properties of FlAsH-EDT_2_ before and after labelling onto the FCM-ybbR peptide, Fluorescence Correlation Spectroscopy (FCS) was performed on a custom built confocal microscope^57^. Experiments were performed using a 488 nm blue laser (TECBL-488nm, WorldStarTech, Canada) with an excitation intensity of ~200 W/cm^2^, in order to avoid laser-induced dark states of the fluorophore. Intensity fluctuations were converted into correlation curves by a hardware correlator (Flex11–8CH, correlator.com) which were fit using a three-dimensional diffusion model with multiple triplet states^27^:

4 $$G(\tau )=\frac{1}{\langle N\rangle }{(1+\frac{\tau }{{\tau }_{D}})}^{-1}\cdot {(1+\frac{1}{{s}^{2}}\frac{\tau }{{\tau }_{D}})}^{-1/2}\cdot \prod _{1}^{i}(1+\frac{{K}_{i}}{1-{K}_{i}}{e}^{-\tau /{t}_{i}})$$

The parameter $\langle N\rangle$ denotes the average number of molecules diffusing through the detection volume with an average diffusion time ${\tau }_{{\rm{D}}}$. The s parameter is an ellipticity factor related to the elongated shape of the confocal detection volume in the axial directions Parameters ${t}_{{\rm{i}}}$ and *K*_i_ are the lifetime and the corresponding population fractions, respectively, of the triplet state *i*. The hydrodynamic radius of the fluorescent diffusing molecule, *R*_*H*_, can be calculated from ${\tau }_{{\rm{D}}}$ using the relation:

5 $$\frac{{{\omega }_{o}}^{2}}{4{\tau }_{D}}=\frac{{k}_{B}\cdot T}{6\pi \eta {R}_{H}}$$

where *ω*_o_ is the width of the confocal detection volume, *k*_*B*_ is the Boltzmann constant, *T* is the temperature, and *η* is the viscosity of the solution.

### TIRF Microscopy

Two-color imaging of FlAsH-FCM-ybbR immobilized on PEG-biotin-neutravidin-coated glass coverslips and labelled *in situ* with CoA-A647 was performed on a custom-built TIRF microscope^57^ The co-localization between FlAsH and AF647 fluorescent molecules was probed using alternating excitation with a 633 nm laser for AF647 fluorescence and a 473 nm laser (Cobolt Blue, Cobolt AB, Sweden) for FlAsH fluorescence. Modulation of laser excitation was achieved using an acousto-optic tunable filter (TF625–350–2–11-BR1A, Gooch & Housego). A long-pass (LP-488-RS, Semrock) and a band-pass filter (HQ535/50, Chroma) were used for FlAsH detection, and a long pass 655 (LP-655-RS, Semrock) was used for AF647 detection. A 50 μM × 50 μM region of the sample was viewed on an electron-multiplied charge-coupled device (EMCCD) (DU-897BV, Andor). Single-molecule TIRF movies consisted of 500 frames with an exposure time of 30 ms/frame. TIRF images were color-coded, normalized, and summed using ImageJ^49^.

### Multiparameter Fluorescence Confocal Microscope

Time-resolved fluorescence decay, anisotropy decay and single-molecule FRET measurements were performed on a custom-built microscope featuring time-correlated single-photon counting (TCSP) and alternating-laser excitation (ALEX)^34^. The donor (FlAsH) was excited at 480 nm by frequency doubling the 960 nm output of a tunable femtosecond laser (Tsunami HP, Spectra Physics, USA) that produces ~100 fs pulses at 80 MHz repetition rate. The acceptor (AF647) was excited using a continuous wave 635-nm laser (World StarTech, TECRL-25GC-635-TTL-A). An acousto*-*optic modulator (Isomet, 1205C-2) alternated blue and red excitation at a 50 μs duty cycle using a function generator (Tektronix, afg3022b). Single-photon avalanche photodiodes (SPAD) with low dark noise converted photons into digital signals which were recorded by a multi-channel time-correlated counting module (PicoHarp300, PicoQuant, Germany). Green (donor) and red (acceptor) fluorescence was split within the emission path using a dichroic mirror (FF640-Di01, Semrock) and band-pass filters (BP520/66 m and HQ685/80, Chroma) in the donor and acceptor emission path, respectively. The donor and acceptor emission paths were further split into their parallel and perpendicular emission components using polarizing beam splitters. All the components in the setup were controlled through a graphical user interfaces in LABVIEW and custom-written MATLAB code was used for analysis^39^.

### smFRET experiments and analysis

The smFRET measurements were performed using a concentration of ~50 pM of sample in PBS (0 M NaCl, pH 7.0), with 20 mM cysteamine and 0.01% Tween20. ALEX-modulated smFRET experiments were performed with donor and acceptor excitation intensities of ~25 kW/cm^2^ and ~12 kW/cm^2^, respectively. The FRET efficiency, *E*, was calculated using the following expression using the number of photons detected in the donor, *I*_*D*_, and the acceptor, *I*_*A*_, channels from bursts of fluorescence intensity:

6 $$E=\frac{{I}_{A}}{{I}_{A}+\gamma {I}_{D}}$$

where the gamma factor, γ, corrects for the difference in detection efficiencies between the two channels and the difference in the fluorescence quantum yields of the two dyes^36^. The gamma factor can be expressed in terms of the detection efficiencies for the acceptor emission, η_Aem_, and for the donor emission, η_Dem_, and the quantum yields of the acceptor, Φ_A_, and the donor, Φ_D_:

7 $$\gamma =\frac{{\eta }_{Aem}{{\rm{\Phi }}}_{A}}{{\eta }_{Dem}{{\rm{\Phi }}}_{D}}$$

The γ value was estimated using a previously published protocol^36^. Briefly, the γ value was first determined at the level of single bursts of fluorescence intensity for a solution containing an equimolar mixture of double-stranded DNA standards with 10 and 17 base-pair (bp) separations between the 5′ and the 3′ end, which were labelled with Fluorescein and AF647, respectively (Integrated DNA Technologies). This mixture gave rise to a mixture of low (17 bp) and high (10 bp) FRET species. The center positions of the low and high smFRET populations were adjusted visually until they both passed through the modified static FRET line^58^. The quantum yields of the dsDNA dye-pair were determined at the ensemble level using the comparative method, as described previously^59^. Using these values, the ratio between the detection efficiencies was determined to be 1.09. The quantum yield of FlAsH-FCMybbR and CoA-AF647 were estimated to be Φ_D_ = 0.40 and Φ_A_ = 0.33, respectively, resulting in a gamma correction factor of γ = 0.91. Background counts were measured from the buffer and were subtracted from the data prior to analysis. Spectral-cross-talk corrections were implemented using information from donor-only and acceptor-only bursts^36,39^.

Single molecules with intensity bursts from both donor and acceptor dyes were identified using ALEX time-tags and accumulated into FRET histograms using a custom MATLAB code^39^. Single-molecule bursts were identified using the ‘MLT’ burst search algorithm^50^. Bursts containing at least M = 10 photons/burst were collected within a time-window of T = 500 μs. The data was then further filtered using a threshold of L = 25 photons/burst to minimize photons collected form background, and only the donor-acceptor dye pairs with an emission stoichiometry, S, between 0.2 to 0.8 were histogrammed for smFRET analysis. The donor-acceptor stoichiometry, S, for each burst was calculated using the following expression,

8 $$S=\frac{{I}_{DD}}{{I}_{DD}+{I}_{AA}}$$

where, *I*_*DD*_, and *I*_*AA*_, are the spectrally corrected intensities (*vide supra*) of the donor after donor excitation and of the acceptor after acceptor excitation, respectively^39^.

Peptides whose acceptor photobleached during the duration of the burst were filtered out by selecting for bursts which had a difference in the mean arrival time between the donor and the acceptor that was greater than 200 μs, as published previously^60^. Donor lifetimes were calculated for each burst using the Maximum Likelihood Estimation (MLE) method^61^.

### Molecular dynamics simulations

The initial starting structure was obtained from molecular modeling using PEP-FOLD^62^. All simulations were carried out using the AMBER14 package using the *ff14SB* force field^63,64^. The systems were immersed in a rectangular box of TIP3P water model with neutralizing concentration of ions^65^. Minimization with positional restraints (25 kcal/mol) on the solutes was carried out for 5000 steps, followed by 5000 steps of unrestrained minimization. The first 2500 steps were of steepest descent and was followed by 2500 steps of conjugate gradient minimization. Subsequently, the systems were heated from 0 to 300 K using the Langevin thermostat in steps of 10 K over the course of 60 ps (NVT) and followed by equilibration for 500 ps (NPT)^66^. We carried out the production runs for 300 ns under NPT conditions.

Simulations were performed under periodic boundary conditions using the particle-mesh Ewald method with a 10 Å cut-off applied for Lennard-Jones interactions^67^. The SHAKE algorithm was used for all bonds involving constrained hydrogen and to allow a longer integration step. All simulations performed out with a time step of 2 fs using the *pmemd* module in AMBER14^63,68^. Adaptive Steered Molecular Dynamics (ASMD) was carried out using a velocity of 10 Å/ns and the reaction coordinate is the end-to-end distance between the terminal CA atoms. All the trajectories were analyzed using the *cpptraj* module in AMBER14. The hydrodynamic radii for FlAsH-FCMybbR were computed using HYDROPRO^43^. Structure images were generated using UCSF Chimera^69^ and PyMOL^70^.

The dipole orientation factor, κ^2^, within the 300-ns simulation time-window was calculated using the distance between the centres of the donor and acceptor dyes, $\mathop{{r}_{da}}\limits^{\longrightarrow}$, and the orientations of the transition dipole moments of the donor, $\vec{d}$, and the acceptor, $\vec{a}$:

9 $${\kappa }^{2}={[\mathop{d}\limits^{\longrightarrow}\cdot \mathop{a}\limits^{\longrightarrow}-3(\mathop{d}\limits^{\longrightarrow}\cdot \mathop{{r}_{da}}\limits^{\longrightarrow})(\mathop{a}\limits^{\longrightarrow}\cdot \mathop{{r}_{da}}\limits^{\longrightarrow})]}^{2}$$

The atomic positions of C12 and C16 defined the vector for the transition dipole moment of FlAsH, whereas the vector joining atomic positions N5 and N4 defined that of AF647^37^.

### Data availability

Datasets generated during and/or analyzed during the current study are available from the corresponding authors upon reasonable request.

## Electronic supplementary material

Supplementary Information

Movie S1

Movie S2

Movie S3

Movie S4
