# Supplementary material for: Characterization of Fluorescein Arsenical Hairpin (FlAsH) as a Probe for Single-Molecule Fluorescence Spectroscopy
Source: Sci Rep. 2017 Oct 12;7:13063. doi: 10.1038/s41598-017-13427-8 (PMC5638890; doi:10.1038/s41598-017-13427-8)
Supplement: Supplementary file 1 — Supplementary Information [file 41598_2017_13427_MOESM1_ESM.pdf]

SUPPLEMENTARY INFORMATION

Characterization of Fluorescein Arsenical Hairpin (FlAsH) as a Probe for Single-Molecule Fluorescence Spectroscopy

AUTHORS:

Dennis D. Fernandes[1,2,4], Jasbir Bamrah[2], Senthilkumar Kailasam[3], Gregory-Neal W. Gomes[1,2], Yuchong Li[1,2], Hans-Joachim Wieden[3], and Claudiu C. Gradinaru[1,2,4]

AUTHOR AFFILIATIONS:

[1]Department of Physics, University of Toronto, Toronto, Ontario, M5S 1A7, Canada; [2]Department of Chemical & Physical Sciences, University of Toronto Mississauga, Mississauga, Ontario, L5L 1C6, Canada. [3]Alberta RNA Research & Training Institute, Department of Chemistry & Biochemistry, University of Lethbridge, Lethbridge, Alberta T1K 3M4. [4] Authors to whom correspondence may be addressed.

SUPPLEMENTARY TABLES

Supplementary Table S1. Time-resolved fluorescence and anisotropy decay fitting parameters using **equations (1) and (3)**, respectively, for FlAsH-EDT$_2$ and FlAsH-FCMybbR in PBS buffer (150 mM NaCl, pH 7.4).[a]

	FlAsH-EDT$_2$	FlAsH-FCMybbR
τ_{L1} (ns)	0.242 ± 0.002	—
τ_{L2} (ns)	1.13 ± 0.01	0.55 ± 0.01
τ_{L3} (ns)	4.2 ± 0.1	4.72 ± 0.01
B_1 (%)	52 ± 1	—
B_2 (%)	35 ± 1	17 ± 1
B_3 (%)	12 ± 1	83 ± 1
ρ_1 (ns)	0.142 ± 0.001	2.29 ± 0.03
ρ_2 (ns)	—	$\rho \to \infty$
A_1 (%)	—	86 ± 1
A_2 (%)	—	14 ± 1
χ^2	1.24	1.08
AIC	1357	533

[a] Error in fitted parameters correspond to the standard deviation from statistical bootstrapping (50 times).

Supplementary Table S2. FCS fitting parameters using **equation (4)** for FlAsH-EDT$_2$, AF488, and FlAsH-FCMybbR in the absence or presence of 2 mM Trolox and 20 mM Cysteamine in PBS buffer (150 mM NaCl, pH 7.4).[a]

			+2 mM Trolox, +20 mM Cysteamine	
	FlAsH-EDT$_2$	AF488	FlAsH-EDT$_2$	FlAsH-FCMybbR
R_H (Å)	9.1 ± 0. 2	6.3 ± 0. 3	6.6 ± 0. 3	12.4 ± 1.2
τ_D (μs)	81 ± 2	67 ± 1	58 ± 1	108 ± 1
t_1 (μs)	3.4 ± 0.1	11 ± 1	9 ± 1	0.37 ± 0.07
t_2 (μs)	28 ± 3	—	—	8 ± 1
t_3 (μs)	—	—	—	36 ± 2
K_1 (%)	42 ± 1	20 ± 1	34 ± 1	10 ± 1
K_2 (%)	21 ± 1	—	—	30 ± 1
K_3 (%)	—	—	—	33 ± 1

[a] Error in fitted parameters correspond to standard fitting errors from the fitting algorithm.

SUPPLEMENTARY FIGURES

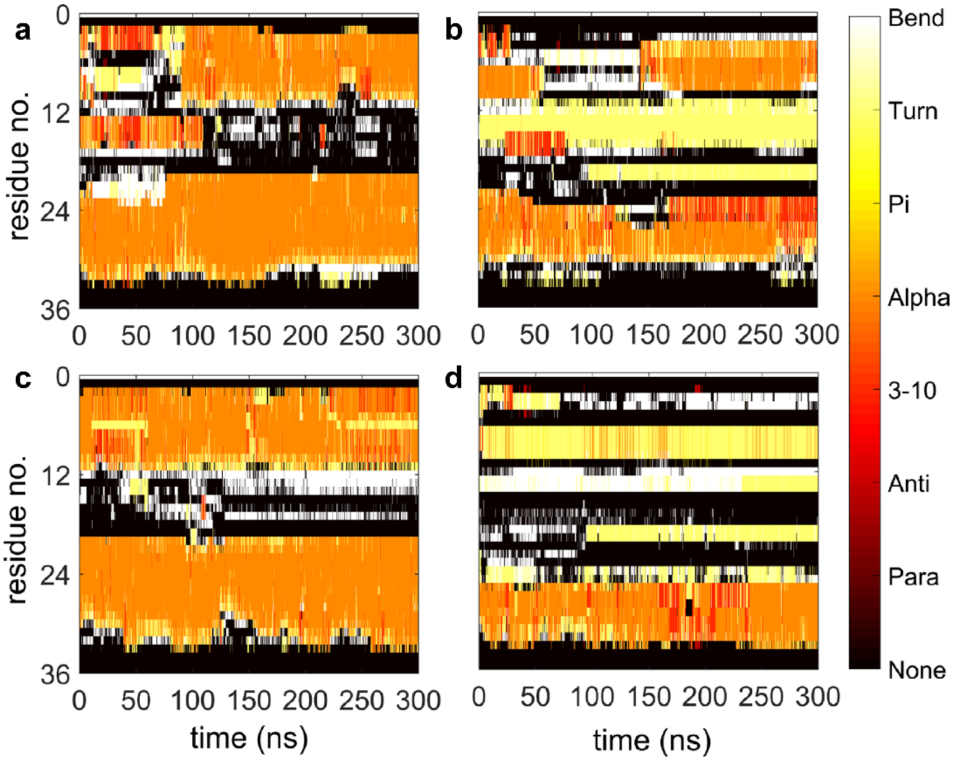

Supplementary Figure S1. Time evolution of the secondary structural elements of the peptide. Heat maps displaying the structural time evolution of **(a)** FCM-ybbR, **(b)** FlAsH- FCM-ybbR, **(c)** FCMybbR-CoA-Alexa647, and **(d)** FlAsH-FCMybbR-CoA-Alexa647. Secondary structure classification is based on database of secondary structure assignments (DSSP) and is depicted by the colored scale bar, varying from black to white.

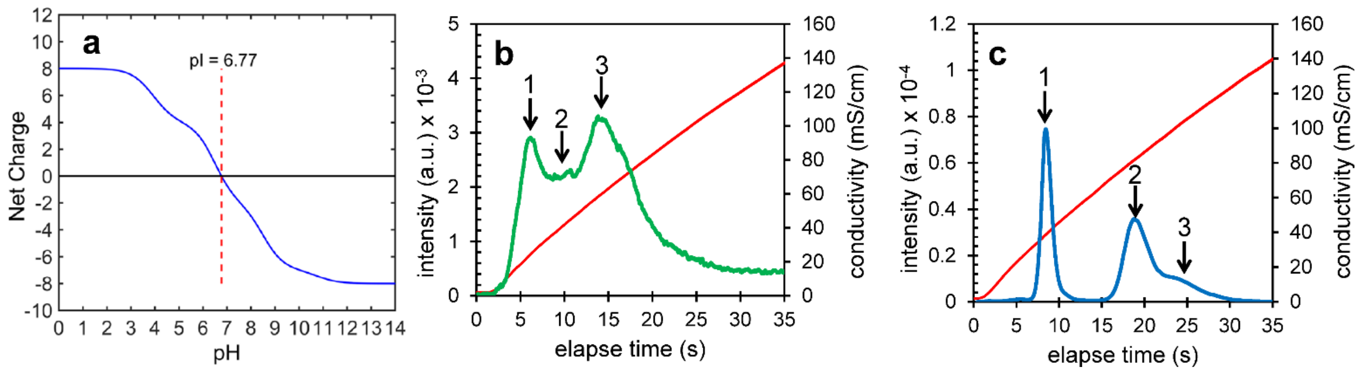

Supplementary Figure S2. Purification of FlAsH-FCM-ybbR and CoA-AF647. (a) The FCMybbR net electrostatic charge as a function of pH, generated using MATLAB code. Dashed red line indicates the isoelectric point, pI= 6.77. **(b)** Anion exchange chromatogram of FlAsH-FCM-ybbR (green) and **(c)** CoA-Alexa647 (blue), superimposed with the respective salt gradients from 0 M to 2 M NaCl (solid red lines). Numbered arrows in **(b)** and **(c)**, identify the eluted peaks chosen for further analysis, as described in the main text. In **(b)**, peaks 1, 2, and 3 correspond to free FlAsH-EDT_2 (-2), unlabeled peptide (-5) and FlAsH-labeled peptide (-7), respectively. In **(c)**, peaks 1, 2, and 3 correspond to free AF647 (-3), unlabeled CoA (-4), and labelled CoA-AF647 (-7), respectively.

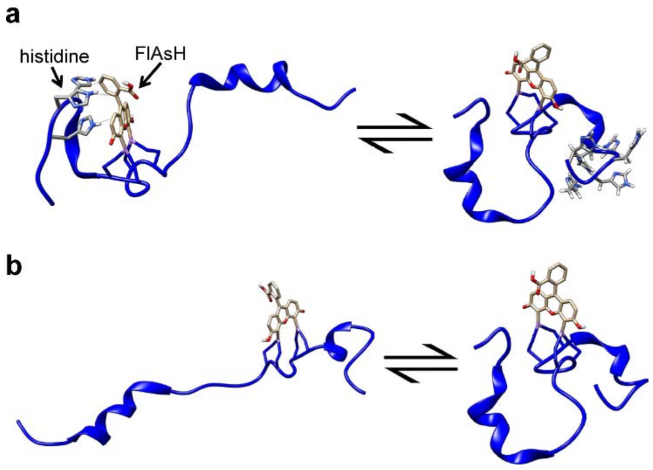

Supplementary Figure S3. Conformational transitions of FlAsH-FCMybbR. (a) Snapshots from MD simulations of FlAsH-FCMybbR illustrating the transition between PET active and inactive states. **(b)** MD snapshots of FCMybbR transitioning from a rod-like conformation to a compact conformation. Timescales of the transitions depicted in **(a)** and **(b)** occur on the order of 100 ns.

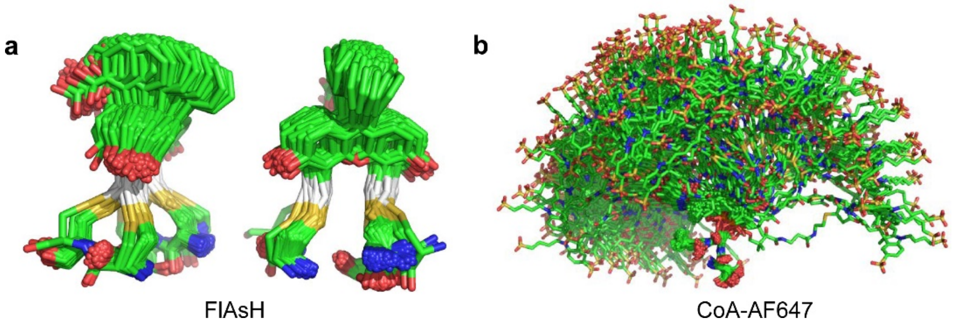

Supplementary Figure S4. Rigidity of FlAsH and rotational-freedom of CoA-AF647. Superimposed rotamers obtained from molecular dynamics simulations for **(a)** FlAsH and **(b)** CoA-AF647, showing the rigidity of FlAsH and the rotational freedom of CoA-AF647.

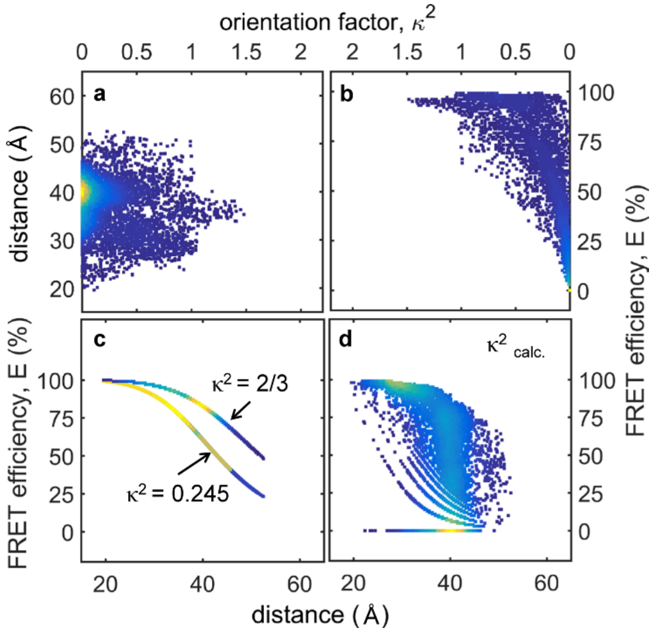

Supplementary Figure S5. Molecular dynamics based FRET computations. Two-dimensional cluster plot of **(a)** the distance between the fluorophores, and **(b)** the corresponding FRET efficiencies as a function of the orientation factor. Computed two-dimensional cluster plots of FRET efficiencies versus distance by **(c)** assuming random dipole orientation between the fluorophore pair, $\langle \kappa^2 \rangle_{iso}$ = 2/3, as well as the time-averaged dipole orientation from MD, $\langle \kappa^2 \rangle_{MDavg}$ = 0.245, and **(d)** from the time-correspondent calculated orientation factor (κ^2 calc.).
